# The Implementation of Industrial Byproduct in Malaysian Peat Improvement: A Sustainable Soil Stabilization Approach

**DOI:** 10.3390/ma14237315

**Published:** 2021-11-29

**Authors:** Afnan Ahmad, Muslich Hartadi Sutanto, Niraku Rosmawati binti Ahmad, Mastura Bujang, Mazizah Ezdiani Mohamad

**Affiliations:** 1 Department of Civil & Environmental Engineering, Universiti Teknologi PETRONAS, Seri Iskandar 32610, Malaysia; muslich.sutanto@utp.edu.my (M.H.S.); niraku.ahmad@utp.edu.my (N.R.b.A.); 2 Civil Engineering Programme, School of Engineering and Technology, University Technology Sarawak, Sarawak 96000, Malaysia; mastura.bujang@ucts.edu.my (M.B.); mazizah@ucts.edu.my (M.E.M.)

**Keywords:** peat, sustainable soil stabilization, eco-friendly, reuse of industrial byproducts, unconfined compressive strength, California Bearing Capacity, scanning electron microscope

## Abstract

Peat is a well-known problematic soil associated with poor engineering properties. Its replacement with an expensive competent foundation material is practiced for road embankment construction which is costly and causes greenhouse gas emissions. Therefore, this paper investigated the effectiveness of a byproduct from a metal industry (silica fume) to stabilize peat along with ordinary Portland cement (OPC) through a series of experimental tests. After peat-indexed characterization, a number of standard compaction and mechanical tests were performed on the stabilized and parent peat. For this purpose, nine designated mixes were prepared possessing various combinations of silica fume (SF) and 10–20% OPC. Unconfined compressive strength (UCS) and California Bearing Ratio (CBR) tests were carried out after 7, 14, and 28 days of curing to assess strength enhancement and binder effectiveness, and the microstructural evolution induced by the binders was examined with scanning electron microscopy (SEM). The analysis revealed a substantial improvement in mechanical properties with the incorporation of SF and OPC, ultimately meeting the minimum strength requirement for highway construction (i.e., 345 kPa). A peak UCS of 1063.94 kPa was recorded at 20% SF, and an unsoaked CBR value of 42.95 was observed using 15% SF and 15% OPC after 28 days of curing. Furthermore, the increasing percentage of hydraulic binders exhibited brittle, collapsible failure, while the microstructural study revealed the formation of a dense matrix with a refined pore structure in the treated peat. Finally, a significant statistical analysis was carried out by correlating the test parameters. In this way, rather than stockpiling and dumping, an industrial byproduct was implemented in peat stabilization in an eco-friendly manner.

## 1. Introduction

Histosol, moss, bogs, fen, and mires are widely classified as peat soil that originates from the anaerobic decaying of plants and animals. Peat soil is considered the most undesirable base material for any sort of construction activity [1]. The poor engineering properties associated with peatland, i.e., high water content and high void ratio, result in low shear strength and compressible behavior, making it unable to bear infrastructure loads. Unluckily, peatland comprises 3% of the world’s land, with Canada and the former USSR being the major contributors; 8% of Malaysia, a tropical country, comprises peatland [2]. However, the rapid growth of Malaysia in Southeast Asia has demanded land acquisition for development projects such as highway construction, housing societies, and industrialization. Thus, the stabilization of peat soil is necessary to attain desirable load-bearing capacities and utilize unserviceable peatland for developing schemes.

Among various soil stabilization techniques, sustainable chemical stabilization gaining interest around the globe [3]. Several binders, including traditional and non-traditional additives, are currently used to strengthen peat [1,2]. However, the implementation of secondary waste materials is highly encouraged to avoid the pressing issues of its disposal [4]. Various raw materials, including scraped tires, demolished concrete waste, silica fume, blast furnace slag, gypsum, oil shale, municipal solid waste, fly ash, and palm oil fuel ashes, have been adopted for the stabilization of peat [2,4]. The implementation of these additives causes physical and chemical changes in peat soil in favor of engineering applications. For instance, Mahmood et al. [5] utilized palm oil fuel ash (POFA) in Malaysian peat at 5%, 10%, 15%, and 20%; an approximate 4-fold increase in the maximum dry density and 31–40-fold-higher CBR values were reported.

Silica fume (SF) is a byproduct derived from the smelting process of silicon, and an alloy containing silicon in an electric arc furnace possesses an extremely fine spherical shape (1/100th of ordinary Portland cement), thus causing health and environmental issues upon dumping and mixing in an open atmosphere. However, its highly amorphous nature with enriched silicon dioxide makes it substantially pozzolanic [6]. The silicon dioxide reacts with the calcium hydroxide in the presence of water to produce calcium silicate hydrate gel, which is responsible for the strength improvement of problematic soil [7]. Therefore, SF has been extensively adopted in the stabilization of peat soil [7,8,9]. For instance, Kalantari et al. [7] utilized SF in the presence of cement to assess the mechanical and compressibility characteristics of peat. The same authors further conducted a comparative study of cement (5–50%) and SF (5–10%) via unconfined compressive strength (UCS) and California Bearing Capacity (CBR) tests in peat stabilization [8]. The effectiveness of adopting SF in the presence of ordinary Portland cement (OPC) for the stabilization of peat has been previously highlighted.

SF and OPC not only bring physical changes but also alter the chemical composition of soil. The micrographs of untreated and cement-, silica-sand-, and kaolin-stabilized peat reported by Wong et al. [10] revealed a clear transformation from loosely packed peat to a compact solid matrix. Moreover, Rikmann et al. [9] performed microstructural testing (XRD, XRF, and FTIR) of the utilization of cement and shale ash in peat stabilization; their results supported the application of pozzolanic additives such as silica fume, pH-modified alkali, and water glass without the addition of ordinary Portland cement for the stabilization of peat soil. In summation, previous findings have shown that both silica fume and OPC are potential binders in peat stabilization.

The stabilization process of soil, especially in the case of peat, is a highly site-specific task because it depends on a number of factors, e.g., peat type, water content, and acidity, as reported by Hebib and Farrell [11]. Additionally, the concentration of humic and fulvic compounds in peat is a key factor that causes an inhibitory effect on the hardening of binder–soil mixtures. However, studies on Peninsular Malaysian peat stabilization via SF and OPC have been limited. The performance of SF and OPC in Peninsular Malaysian peat soil has been exclusively assessed through mechanical testing. Thus, an inclusive mechanical and microstructural assessment of Peninsular Malaysian peat utilizing SF and OPC for soil stabilization is needed to better predict the engineering behavior of the stabilized peat matrixes. Furthermore, comparative analyses of the binder’s effectiveness in peat stabilization are lacking.

In the current research work, the chemical stabilization of Malaysian peat soil was carried out through a series of compaction and mechanical tests, and the morphological characteristics were investigated via scanning electron microscopy (SEM) to examine and compare the microstructural evolution of the parent and stabilized peat matrixes caused by SF and OPC. The mechanical assessment involved a UCS test followed by failure pattern examination and a CBR test in light of the highway construction criterion. Furthermore, strong statistical correlations were examined to investigate the effect of one parameter on the others. Overall, the effectiveness of silica fume with and without cement was assessed in the context of Peninsular Malaysian peat enhancement via mechanical and microstructural approaches.

## 2. Materials and Experimental Procedure

### 2.1. Peat Collection

The peat soil utilized for the experimental investigations was collected from the Kampung Baru, Teluk Intan, Perak state of Peninsular Malaysia. The coordinates of the peat soil sampling were 4°00′16.1″ N, 101°11′11.0″ E, and peat sampling was carried out at 5 feet (1.52 m) of depth. The peat’s chemical composition by percentage weight is illustrated in Table 1.

### 2.2. Silica Fume and OPC (Binders)

The silica fume, obtained from OM Materials (Sarawak) Sdn Bhd as a byproduct, was used as a potential binder in the Peninsular Malaysian peat stabilization. Silica fume is an industrial waste obtained as a secondary material from silicon and ferroalloy production. Moreover, it has a spherical shape with a diameter of approximately 150 nm (100 times smaller than cement granules). Additionally, it has a high pozzolanic potential and is widely used in high-performance concrete. The results of XRF analyses (chemical composition) of SF and OPC by percentage weight are shown in Table 1, and Table 2 illustrates the physical and chemical properties of the silica fume. Commonly available grade 53 OPC was utilized as an additional additive.

### 2.3. Mix Design

Silica fume is a pozzolanic and industrial waste utilized for the stabilization of Peninsular Malaysian peat. The findings of Kalantari et al. [8] suggested that an effective SF content for peat with its natural moisture content is 20% by weight. In this study, three mixes containing SF in varying percentages (10, 15, and 20%) by weight of dry mass were taken into consideration [12], as shown in Table 3. Several trail mixes containing OPC and SF in varying amounts ranging from 5 to 15% were employed to stabilize Teluk Intan peat while keeping the cumulative binder amount below or equal to 20%, as shown in Table 3. For the sake of comparison, trial mixes of 15% OPC alone and in combination with 15% SF were used.

### 2.4. Specimen’s Preparation and Curing

Initially, the indexed properties of Teluk Intan peat were determined using disturbed soil collected from the sampling site. Subsequently, a series of standard compaction, UCS, and CBR tests were carried out on the treated and untreated peat. The compaction tests were performed on a peat sample passed through a No. 4 sieve utilizing a 101.6 mm (4 in) diameter mold. Similarly, the specimen preparation for UCS testing was carried out according to ASTM D2166/D2166M-16 [13]. As prescribed by ASTM guidelines, all specimens were molded to keep a height to diameter ratio of 2:1. Each UCS sample was prepared by tamping a mass of peat mixed with OMC into three layers in a cylindrical tube with a diameter equal to 35 mm; each layer received 27 blows [10]. All molds were disclosed after one day, exposing the untreated peat to compressive testing while keeping the treated samples for 7, 14, and 28 days of curing. The curing technique prescribed by Kaniraj and Gayathri [14] was followed. All the stabilized UCS samples were carefully wrapped in airtight plastic bags and kept in a controlled environment for 7, 14, and 28 days of curing to avoid moisture loss and maintain temperature (25 °C). A similar curing procedure was adopted for CBR samples; following ASTM D 1883-16, using a mold with a diameter of 152.4 mm (6 in) and a height of 101.6 mm (4 in) [15].

### 2.5. Experimental Testing Matrix

The experimental procedure of peat stabilization was initiated with a basic properties’ examination. These indexed properties play vital roles in peat characterization and may hinder the stabilization process. Table 4 illustrates the entire experimental matrix including standard compaction, UCS, and CBR. As illustrated in Table 4, a total of 9 (one parent and 8 treated peat) designated mixes were tested. Three trials of every test was conducted, and the averages of three tests are presented as the outcomes.

#### 2.5.1. Standard Compaction

Standard compaction tests of the treated and untreated Teluk Intan peat were carried out according to ASTM D698-12 [16]; soil samples were subjected to about 600 kN-m/m^3^ (12,400 ft-lbf/ft^3^) over three layers, and maintained 25 blows per layer using a 24.5 N (2.5 kg) rammer dropped from a height of 305 mm (12 in). Cumulatively, 27 standard compaction tests were carried out on the 9 designated mixes by performing 3 trials on every mix.

**Table 4:** Standard compaction and mechanical property testing matrix.

Mixes	Standard Compaction	UCS			CBR					
					Soaked			Unsoaked		
		7 D ^1^	14 D ^1^	28 D ^1^	7 D ^1^	14 D ^1^	28 D ^1^	7 D ^1^	14 D ^1^	28 D ^1^
Peat	✓	-	-	-	-	-	-	-	-	-
Peat and 10% SF	✓	✓	✓	✓	✓	✓	✓	✓	✓	✓
Peat and 15% SF	✓	✓	✓	✓	✓	✓	✓	✓	✓	✓
Peat and 20% SF	✓	✓	✓	✓	✓	✓	✓	✓	✓	✓
Peat and 15% OPC	✓	✓	✓	✓	✓	✓	✓	✓	✓	✓
Peat, 15% SF, and 15% OPC	✓	✓	✓	✓	✓	✓	✓	✓	✓	✓
Peat, 5% SF, and 15% OPC	✓	✓	✓	✓	✓	✓	✓	✓	✓	✓
Peat, 10% SF, and 10% OPC	✓	✓	✓	✓	✓	✓	✓	✓	✓	✓
Peat, 15% SF, and 5% OPC	✓	✓	✓	✓	✓	✓	✓	✓	✓	✓

^1^ D denotes days of curing period; ✓: Tests performed.

#### 2.5.2. Unconfined Compressive Strength (UCS)

The UCS tests on the parent peat were conducted after one day of sampling, while the stabilized samples were exposed to the UCS test after curing periods of 7, 14, and 28 days. About 72 (24, 24, and 24) total stabilized UCS samples were tested after 7, 14, and 28 day curing periods, as described in Table 4. ASTM D2166/D2166M-16 [13] was followed for UCS testing.

#### 2.5.3. California Bearing Ratio (CBR)

Like UCS, the CBR tests of the parent peat were carried out after one day of curing, while the stabilized, unsoaked specimens were exposed to load penetration after 7, 14, and 28 days of curing. The stabilized, soaked peat CBR samples were placed in the water bath for 4 days after curing before being subjected to load penetration. Collectively, 144 cumulatively (72 soaked and 72 unsoaked) stabilized CBR samples were tested after 7, 14, and 28 days of curing, as illustrated in Table 4. ASTM D 1883-16 [15] was followed for the CBR testing procedure.

#### 2.5.4. Scanning Electron Microscopy (SEM)

SEM was used to assess and compare the morphology of the treated and parent Teluk Intan peat. The SEM machine (Zeiss EVO LS 15, Seri Iskandar, Perak, Malaysia) available in the Universiti Teknologi Petronas was utilized for all micrographs. The untreated peat and stabilized peat cured for 28 days were SEM-imaged at magnifications of 1000×–10 k×.

## 3. Results and Discussion

### 3.1. Peat Indexed Properties

Peat soil is predominantly composed of organic and mineral components. The first component is carbonaceous in origin and combustible in nature, and the mineral constituents form ash after burning due to their incombustible nature. Both these constituents play vital roles in classification and influence peat behavior [17]. The experimental findings indicated that Teluk Intan peat contains 80.86% of organic content and 19.13% of ash content, as presented in Table 5. Thus, it is clear that the collected sample possessed more than 75% of organic matter, so they could be termed “peat” according to ASTM D 4427–13 [18,19]. Additionally, the peat samples were classified as having high ash contents according to ASTM D2974-00 [20]. Both the reported organic and ash contents were in agreement with previously reported studies on Peninsular Malaysian peat soil [21,22]. Moreover, the collected peat was blackish-brown in color ,and the in situ von Post humification test categorized the Teluk Intan peat as fibric peat (H_3_). The fiber content test showed an 80.4% value and verified the samples as fabric peat. The permeability test revealed high fiber content and specific gravity (1.88), which were directly related to the samples’ void spaces, as shown in Table 5.

### 3.2. Standard Compaction

The application of hydraulic binders, e.g., SF and OPC, alters the properties of peat. It has significant effects on the density of a treated peat matrix and the water amount required to attain the maximum dry density (MDD), commonly called the optimum moisture content (OMC) [28]. In this light, standard compaction tests were carried out for all the designated combinations of peat and additives to assess their effects on MDD and OMC.

**Figure 1:**
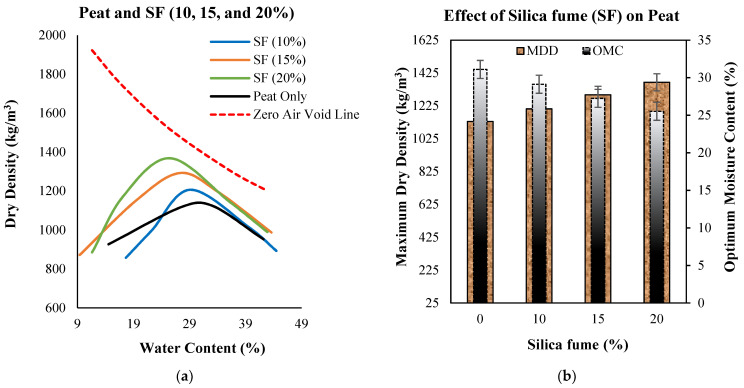
(**a**) Standard compaction curves of untreated and treated peat with 10%, 15%, and 20% SF; (**b**) effects of increasing SF dosage on the dry density and W_opt_ of treated peat.

**Figure 2:**
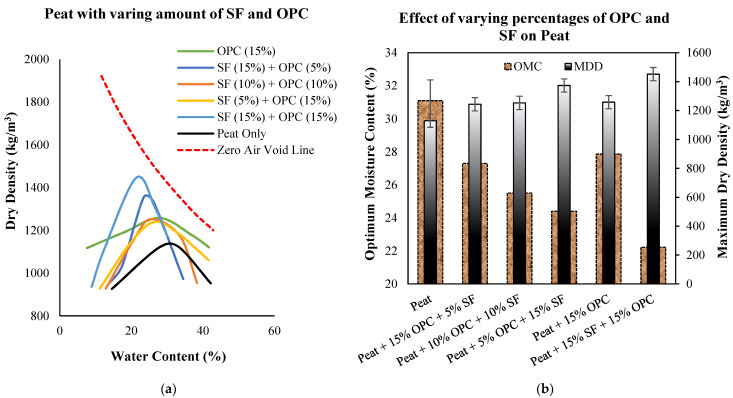
(**a**) Standard compaction curves of untreated and treated peat with varying percentages of SF and OPC; (**b**) effects of varying SF–OPC dosages on the dry density and W_opt_ of treated peat.

The compaction curves of untreated Teluk Intan peat and peat treated with 10%, 15%, and 20% silica fume (SF) are shown in Figure 1a. The MDD and OMC (W_opt_) of untreated Teluk Intan peat were recorded at about 1130 kg/m^3^ and 31.1%, respectively. Figure 1b indicates the effects of different percentages of SF on W_opt_ and MDD. It can be observed that the dry density increased and OMC (W_opt_) decreased as the amount of silica fume increased. Furthermore, Figure 2 indicates the combined effect of SF and OPC on the compaction curves. It can be observed that the incorporation of SF tended to better reduce the OMC and increase the MDD compared to OPC. This may have been due to the SF particles’ fineness and greater surface areas for water absorption. However, all effects of OPC and SF on compaction characteristics are associated with their finer and adhesive natures, which filled up the pores of peat soil and bound the particles together to make a dense matrix, thus increasing the density of the stabilized peat compared to untreated peat. Meanwhile, the lowering of OMC was due to the hydraulic reactions of OPC and SF. The involvement of hydraulic reactions consumed water and subsequently generated heat, so the consumption of water in the peat reduced the optimum water content [29]. In consideration of the swampy environment of peatland, hydraulic binders are suitable for consuming water during the hydration process, thus enhancing peat strength. Similar results have been reported in previous studies on peat stabilization using cement and silica [7,30,31].

### 3.3. Mechanical Testing

#### 3.3.1. UCS Development

**Figure 3:**
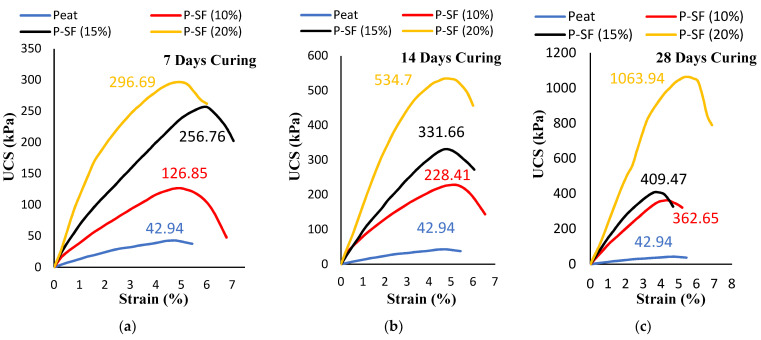
The unconfined compression strength of 10%, 15%, and 20% SF-stabilized peat under varying curing periods: (**a**) 7 days, (**b**) 14 days, and (**c**) 28 days.

**Figure 4:**
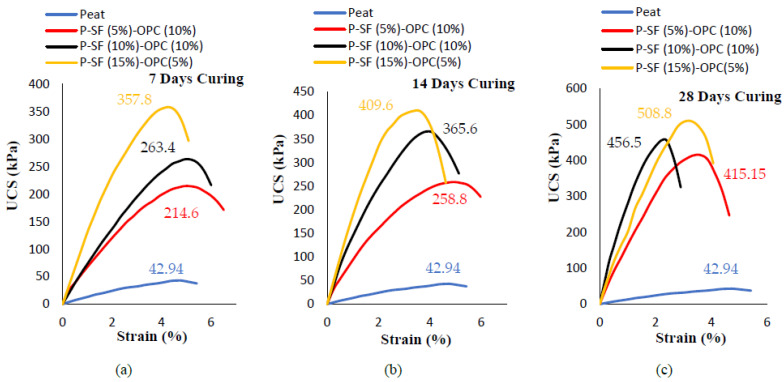
The unconfined compression strength of SF- and OPC-stabilized peat under varying curing periods: (**a**) 7 days, (**b**) 14 days, and (**c**) 28 days.

**Figure 5:**
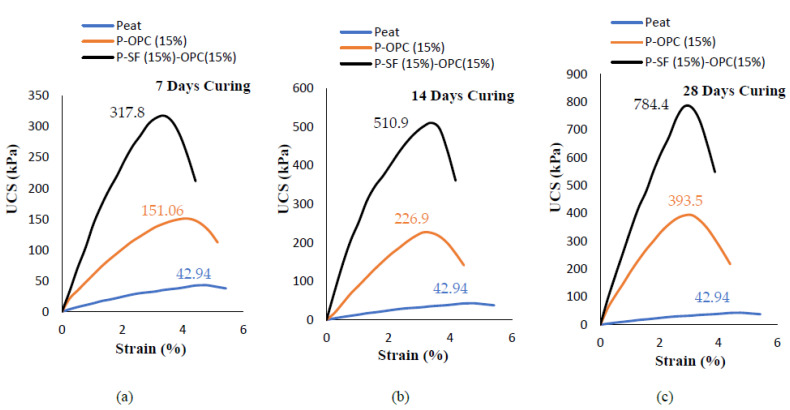
The unconfined compression strength of untreated peat, 15% OPC-stabilized peat, and 15% SF- and 15% OPC-stabilized peat under varying curing periods: (**a**) 7 days, (**b**) 14 days, and (**c**) 28 days.

The UCS results of untreated peat and 8 designated treated mixes are presented in Figure 3, Figure 4 and Figure 5. The untreated Teluk Intan peat showed a low strength of about 42.94 kPa due to the high amount of organic matter, which imparted compressible and collapsible characteristics in the parent peat, thus yielding a low compressive strength—as also stated in previous studies [32,33,34].

Various factors, including the type of peat, water content, mineral content, organic, content, fiber content, and pH, affect the strength gain of cement-stabilized peat. The UCS results of the Teluk Intan peat stabilized with varying SF contents (10, 15, and 20%) and cured for 7, 14, and 28 days are presented in Figure 3a–c. it can be observed that the compressive strength significantly increased as the amount of SF and curing period increased. The highest strength was recorded by mixing 20% SF in all curing periods. The highest values of UCS after 7, 14, and 28 days of curing were 296.6, 534.7, and 1063.94 kPa, respectively, which also indicated the development of strength as the curing duration was prolonged. The strength enhancement of stabilized peat using silica fume was due to the formation of calcium silicate (C–S) and calcium silicate hydrate (C–S–H) gels [35]. Similarly, the effectiveness of OPC in peat soil has been previously reported [8,36,37]. However, the strength enhancements of SF- and OPC-stabilized peat with curing were different due to the differences in hydration rate. Comparatively, SF led to higher strength enhancements than OPC due to peat’s acidic nature, which hindered the stabilization caused by OPC.

Additionally, the detrimental environmental effect of OPC has hindered its applications. For the sake of comparison, OPC was replaced by different amounts of SF for peat stabilization, as shown Figure 4 and Figure 5. To assess the combined effect of SF and OPC, three different mix combinations were made: 5% SF and 15% OPC, 10% SF and 10% OPC, and 15% SF and 5% OPC. The cumulative quantity of binders was kept at 20% because the highest strength of Teluk Intan peat was achieved with 20% SF content. Moreover, Kalantari et al. [8] suggested a 20% cumulative OPC and SF binder for peat stabilization. Figure 4a–c shows the 7, 14, and 28 day curing-strengths of SF (5, 10, and 15%) and OPC (5, 10, and 15%), respectively. Figure 5**.** indicates the strength obtained by using 15% SF, as advised by Kalantari et al. [8], and a trail mix of adopting 15% OPC and 15% SF. It can be observed that the achieved strength was higher than the parent peat due to its fine pozzolanic nature. However, the gained strength was still lower than the strength obtained when using 20% SF. Comparatively, the strength gained by using SF was higher compared to that gained by using OPC; this may have been due to the extremely fine size of silica fume, which is also approximately 120–200% more pozzolanic than OPC [35]. Moreover, the presence of humic acid in peat soil reduces the efficiency of OPC by retarding strength enhancement. For this reason, Axelsson et al. [38] suggested utilizing a surplus amount of OPC to neutralize the humic acid. Thus, using SF (a waste material and effective binder) to enhance the strength of peat is a more cost-efficient and sustainable solution than using OPC. Combining SF and OPC for strength development is associated with pozzolanic reactions and the development of calcium silicate (CS), calcium aluminate hydrate (C–A–H), calcium silicate hydrate (C–S–H) bonds, and ettringite (AFt) formation [7,8,39].

#### 3.3.2. Strength Development by Various Mix Designs

**Figure 6:**
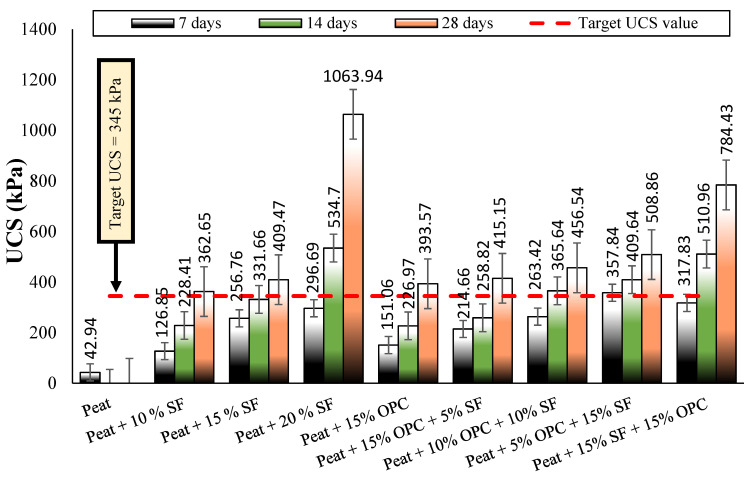
Comparing the compressive strength of untreated peat and stabilized peat after 7, 14, and 28 days of curing.

ASTM D 4609 (Standard Guide for Evaluating Effectiveness of Admixtures for Soil Stabilization) specifies a UCS value of 345 kPa for a stabilized soil to be considered an effective binder, as shown in Figure 6 [29]. As such, the strength development index (SDI) obtained with Equation (1) is used, to assess the influence of various mix designs on peat strength [40].

(1) $$\mathrm{SDI}=\frac{\mathrm{Max}.\;\mathrm{UCS}\;\left(\mathrm{stabilized}\right)-\;\mathrm{Max}.\;\mathrm{UCS}\;\left(\mathrm{parent}\right)}{\mathrm{Max}.\;\mathrm{UCS}\;\left(\mathrm{parent}\right)}$$

where Max. UCS_(stabilized)_ and Max. UCS_(parent)_ indicate the ultimate unconfined compressive strength of treated and untreated peat, respectively.

**Figure 7:**
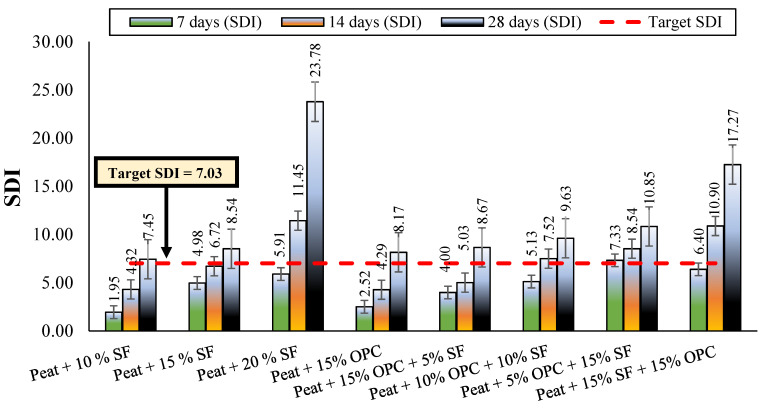
Strength development index (SDI) of all mixes after 7, 14, and 28 days of curing.

Figure 7 shows the SDI results of stabilized and parent peat cured for 7, 14, and 28 days. Based on the untreated peat UCS value (42.94 kPa) and the targeted UCS value (345 kPa), an SDI of 7.03 was calculated, as indicated in Figure 7. It was observed that the parent peat and almost all stabilized peat cured for 7 days fell below the targeted unconfined compression strength. After extending the curing period to 14 days, several mixes met the minimum strength criteria: peat and 20% SF; peat, 10% SF, and 10% OPC; peat, 5% OPC, and 15% SF; and peat, 15% OPC, and 15% SF. All the stabilized peat mixes met the minimum UCS criteria advised by ASTM D 4609 after 28 days of curing, as shown in Figure 6 and Figure 7. A slower development of UCS when using OPC compared to when using SF was observed here and previously in [41]. Considering the targeted SDI value of 7.03, both OPC treated peat cured for 28 days and SF treated peat cured for 14 days encouraged subgrade improvement.

#### 3.3.3. Binder’s Effect on the Failure Modes

**Figure 8:**
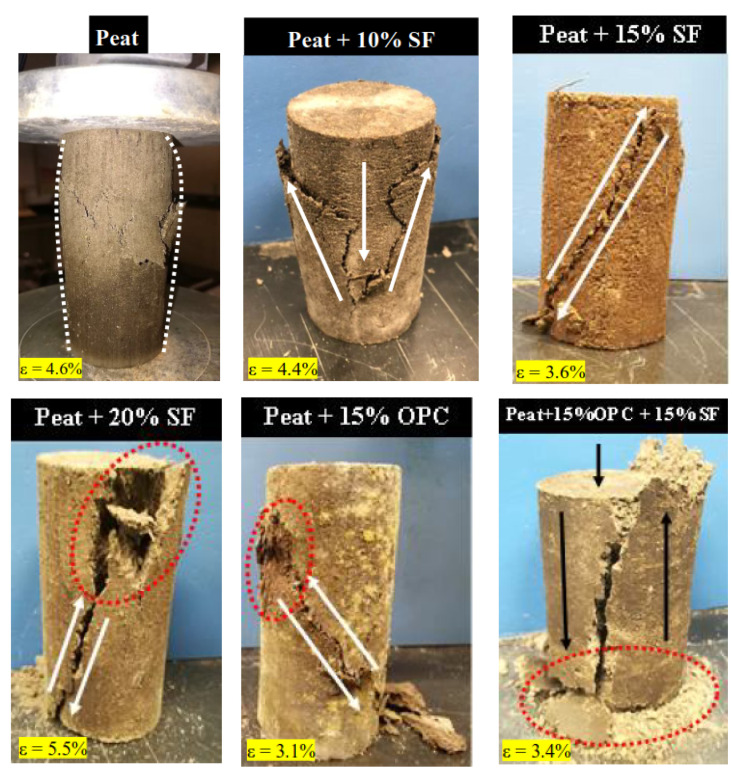

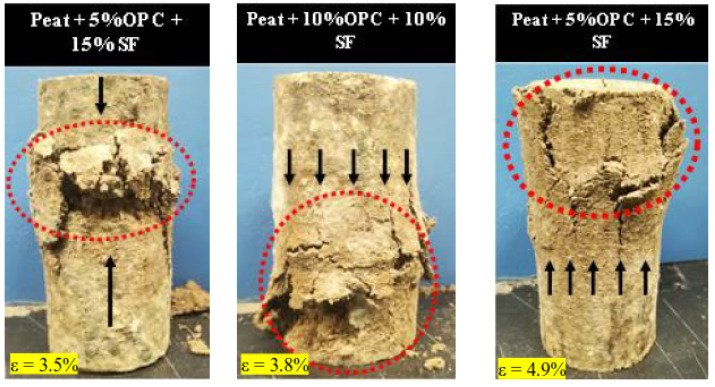
Failure modes of the stabilized and parent peat.

The presence of fibers and partially decomposed vegetation induces structural anisotropy in peat, thus affecting the compression modes of failure [42]. The failure modes of the parent Teluk Intan peat and stabilized peat are presented in Figure 8. It can be observed that the failure pattern changed with the stabilization of peat. Moreover, the failure behavior changed with binder dosages due to changes in density [43].

Due to the high fiber and organic content, the parent peat underwent bulging with an unrecognized failure plane upon axial compressive loading, as shown in Figure 8. However, it was observed that the utilization of hydraulic binders increased stiffness and thus led to a change in the failure mode. This type of failure mode is associated with ductile behavior [44]. The bulging failure mode subsided, and a compressive V-shaped failure pattern was observed in the 10% SF and 15% OPC treated UCS samples, which indicated a reduction in the ductility with the use of a hydraulic binder. On the other hand, a clear shear failure pattern was noticed in the 15% SF treated peat due to its increased stiffness causing brittleness. In the same way, steep sheerness and high brittleness with bursting failure, causing the destruction of the entire UCS specimen, were observed in the 20% SF treated peat and the 15% SF and 15% OPC treated peat, as shown in Figure 8. This means that the incorporation of higher hydraulic binders caused a predominant failure plane that induced brittleness. This brittle behavior has been previously reported to be caused by increasing amounts of hydraulic binder [44,45]. Additionally, non-recognizable compressive collapsible failure was noticed in the 5% OPC and 15% SF, 10% OPC and 10% SF, and 15% OPC and 5% SF treated peat, as shown in Figure 8, indicating its structural heterogeneity. Thus, additives such as fibers, shredded tires, and geofoam materials are recommended in tandem with hydraulic binders in peat to limit brittle and collapsible failure.

#### 3.3.4. CBR and Comparison

The CBR test is an essential test conducted to estimate the shearing resistance (strength) of a subgrade or subbase of pavement. It is used to examine the durability, efficiencies, and thus overall performance of a pavement foundation material. The subgrade can either be a natural existing ground (original subgrade) or an embankment constructed from borrowed material (new subgrade). In both cases, the minimum permissible CBR value for subgrade is 10% [46], and the relative ratings of the shearing resistance based on CBR values are given in Table 6 [47].

**Figure 9:**
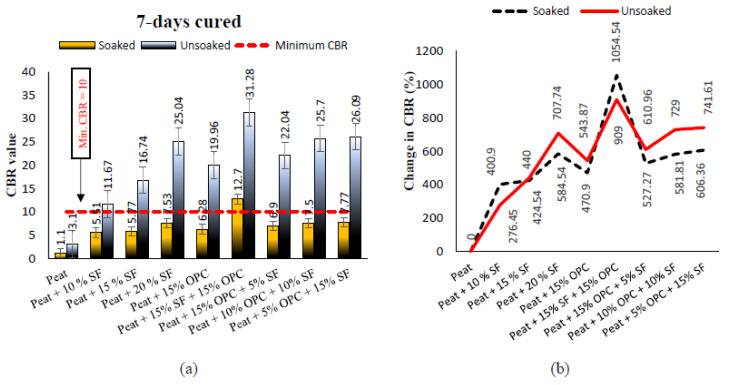
(**a**) The 7 days cured soaked and unsoaked CBR values of treated and untreated Teluk Intan peat; (**b**) changes in soaked and unsoaked CBR values after treatment.

**Figure 10:**
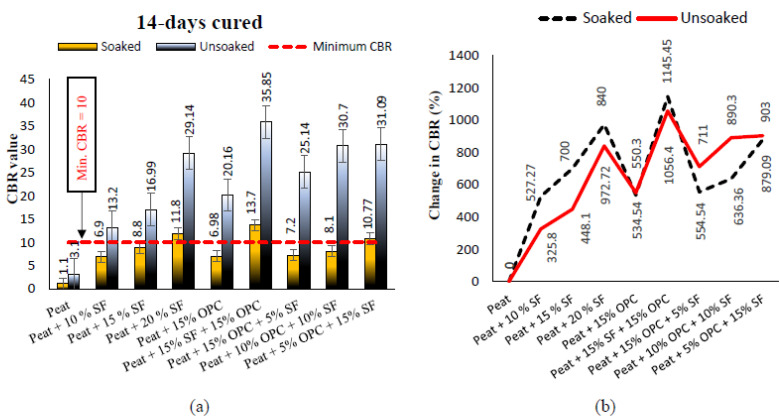
(**a**) The 14 days cured soaked and unsoaked CBR values of treated and untreated Teluk Intan peat; (**b**) changes in the soaked and unsoaked CBR values after treatment.

**Figure 11:**
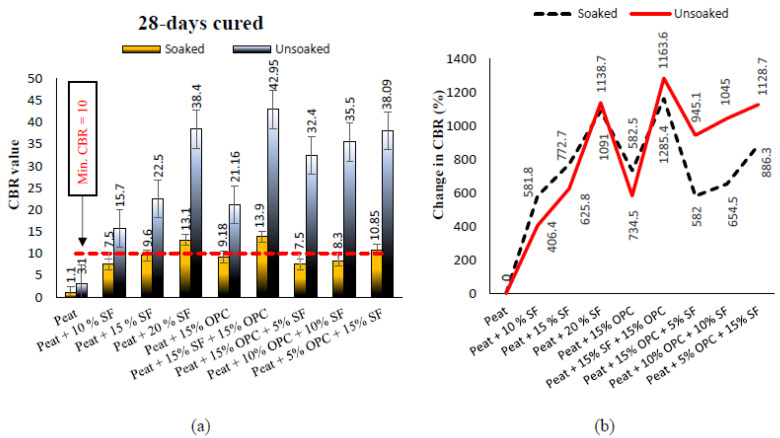
(**a**) The 28 days cured soaked and unsoaked CBR values of treated and untreated Teluk Intan peat; (**b**) changes in the soaked and unsoaked CBR values after treatment.

The soaked and unsoaked CBR values of treated and untreated Teluk Intan peat cured for 7, 14, and 28 days are presented in Figure 9, Figure 10 and Figure 11, respectively. The parent Teluk Intan peat showed extremely low soaked and unsoaked CBR values of 1.1% and 3.1%, respectively. In the literature, the unsoaked CBR value of undisturbed peat has been shown to be as low as 0.785% [48] and ranging from 2.43 to 5.66% [49,50,51]. The Table 6 ratings suggest that Teluk Intan peat very poor in strength for subgrade materials and need to be treated to enhance their shearing resistance.

The treatment of Teluk Intan peat with hydraulic binders (SF and OPC) showed substantial improvements of the CBR value after curing for 7, 14, and 28 days. As noticed in Figure 9a, all the designated mixes obtained a higher unsoaked CBR value than 10 after 7 days of curing, which indicated their suitability as subgrade materials. The highest CBR value of the specimens cured for 7 days specimens was obtained by treating peat with 15% SF and 15% OPC, as shown in Figure 9b. Similarly to UCS, CBR was increased after prolonging the curing period due to pozzolanic reactions, as shown in Figure 10 and Figure 11. Moreover, the CBR of the stabilized peat increased with the increasing SF and OPC contents. Even the soaked samples of peat; 20% SF and peat; and 15% OPC, and 15% SF achieved CBR values of more than 10, indicating their feasibility as subgrade materials. The observed strength enhancement due to SF may be associated with the formation of calcium silicate hydrate (C–S–H) gel and calcium silicate (CS) [7,52], while the combined effect of SF and OPC may be associated with the formation of calcium silicate (CS) and hydrated components as a result of hydration, i.e., calcium aluminate hydrate (C–A–H), calcium silicate hydrate (C–S–H) gel, and AFt formation. [1]. Moreover, the development of greater interfacial confinement bonding, roughness, contact area, and friction mobilization upon the loading of the stabilized peat yielded higher CBR values [53]. Comparatively, the strength gains caused by the combined SF and OPC was higher than the other treatments. About 1054%, 1145%, and 1163% CBR was recorded by 15% SF and 15% OPC treated peat after curing for 7, 14, and 28 days of curing, respectively. However, OPC is expensive and causes environmentally hazardous effects, so its adoption is discouraged. Therefore, SF, which is an industrial by-product waste that is cementitious, is a more sustainable and cost-effective binder than OPC.

### 3.4. Morphological Variations

SEM was used to reveal the morphology of the parent and treated Teluk Intan peat. The internal mineralogical formation upon the application of hydraulic binders, i.e., silica fume (SF) and OPC, significantly alter the strength of the peat soil.

**Figure 12:**
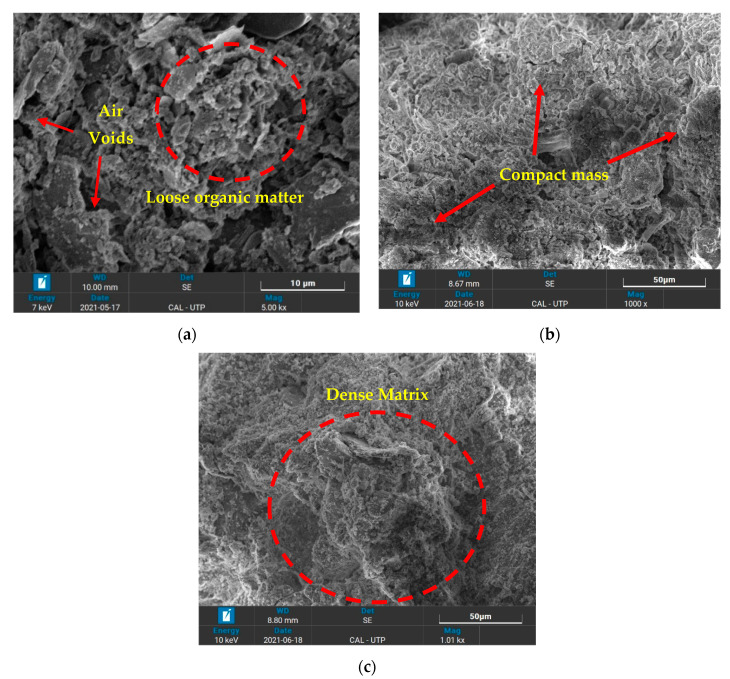
Micrographs of treated and untreated peat: (**a**) peat; (**b**) peat and SF; and (**c**) peat, SF, and OPC.

It can be observed in Figure 12a that the internal structure of the parent peat consisted of hollow cavities/pores that were flaky and loosely packed, as well as spongy organic matter. Typically, organic matter is hollow and spongy in nature, so they possess a high water-holding capacity upon saturation [10,33]. The same morphology of Malaysian peat has been reported by other researchers [10,30,54,55,56,57]. These are the features of parent Teluk Intan peat that are responsible for its low UCS and CBR values, as observed during the mechanical testing. On the other hand, a more dense and compacted microstructure compared to parent peat was observed in the 28 days cured SF peat and the SF- and OPC-stabilized peat, as shown in Figure 12b,c, respectively. The formation of hydration products, i.e., C–S–H gel, C–A–H, and Aft, caused a reduction in the micropores, thus yielding a dense and compact matrix of peat that reasonably enhanced its strength [54,58,59].

### 3.5. Statistical Correlations

To elucidate the interdependency of SF-stabilized peat soil parameters, the statistical correlations among the UCS, CBR (soaked and unsoaked), and SF content were evaluated. The use of correlations is a well-established and widely adopted technique to investigate the effect of one parameter on others. Moreover, it can be used to indicate the closeness of experimentally investigated values to a tailored trend (regression) line that ranges from 0 to 1 via the coefficient of determination (R^2^). Generally, any regression model that possesses an R^2^ value equal to or close to 1 is considered a more effective predictive model than one near zero. Several studies have adopted the statistical correlation method to identify the effect of one parameter on others [60,61,62].

#### 3.5.1. Compaction Parameters Correlations

**Figure 13:**
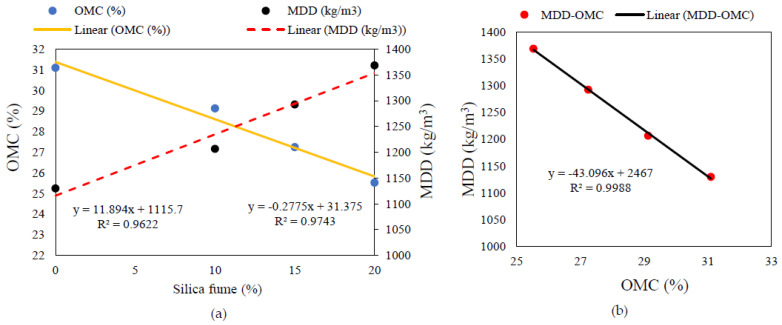
Statistical correlation among the peat compaction parameters: (**a**) variation of OMC and MDD with SF content and (**b**) variation of MDD with the OMC of SF-stabilized peat.

The OMC and MDD have significant effects on the engineering properties of soil. A highly significant statistical correlations (R^2^ > 0.96) among the compaction parameters and the increasing amount of silica fume, as shown in Figure 13. In Figure 13a, two linear significant regression models can be seen, thus indicating an increasing trend in the maximum dry density and a decreasing trend in the OMC with the increased amount of SF content. Moreover, a significant correlation among the OMC and MDD of SF-stabilized peat were observed in the range of 10–20% of silica fume content, as illustrated in Figure 13b. It can be seen that MDD predominantly decreased with the increasing amounts of OMC, which could also be clearly observed in the mechanical characteristics.

#### 3.5.2. Mechanical Strength Parameters and Binders Dosage Effects

**Figure 14:**
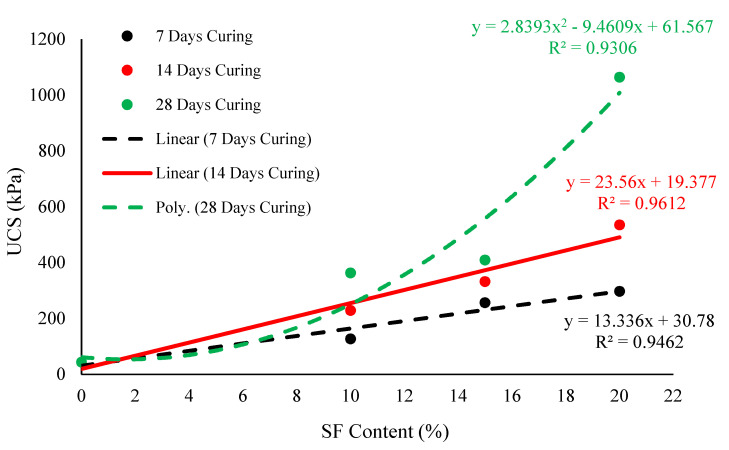
The statistical relationship between the increasing amounts of SF and UCS obtained after 7, 14, and 28 days of curing.

**Figure 15:**
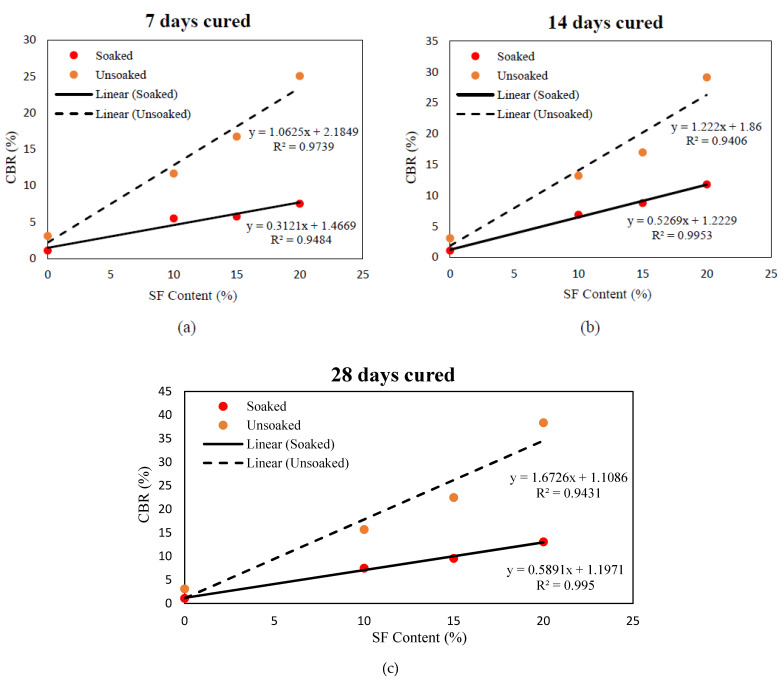
Statistical relationship between increasing amounts of SF and CBR (soaked and unsoaked) obtained after 7 (**a**), 14 (**b**), and 28 (**c**) days of curing.

Binder content plays a vital role in peat stabilization, as observed from our mechanical property testing and previous studies. Figure 14 and Figure 15 illustrate the statistical correlations among UCS–SF, CBR–SF, and UCS–CBR content, respectively. Figure 14 shows a strong quadratic model (R^2^ > 0.90) among the UCS and SF contents for the 7, 14, and 28 day curing periods. Using Equations (2)–(4), the unconfined compression strength was predicted for the stabilized peat cured for 7, 14, and 28 days, respectively.

(UCS)_7days_ = 13.336 × (SF) + 30.78 (2)

(UCS)_14days_ = 23.56 × (SF) + 19.377 (3)

(UCS)_2 days_ = 2.8393 × (SF)^2^ − 9.4609 × (SF) + 61.567 (4)

**Figure 16:**
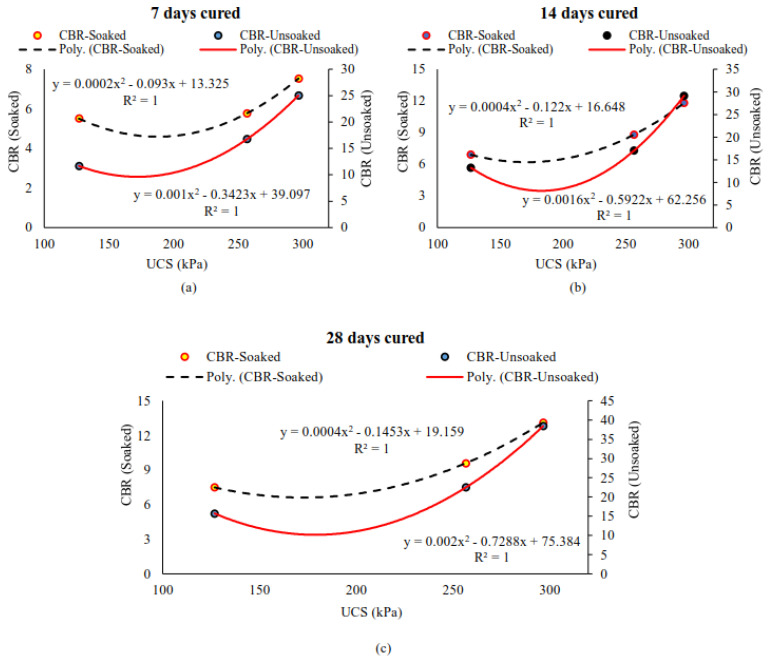
The statistical relationship between UCS and CBR (soaked and unsoaked) obtained after 7 (**a**), 14 (**b**), and 28 (**c**) days of curing.

Similar to UCS–SF content relationship, effective models were developed to predict the CBR values (soaked and unsoaked) of varying SF contents after curing stabilized peat for 7, 14, and 28 days, as shown in Figure 15a–c. Likewise, the regression models shown in Figure 16a–c were developed among the UCS and CBR (soaked and unsoaked) of 10–20% SF-stabilized peat after 7, 14, and 28 days of curing. All the developed models were statistically significant and could be adapted to predict the UCS and CBR values of stabilized peat that incorporates 10–20% of SF. 

Moreover, the statistical mean differences among the soaked and unsoaked CBR values after 7, 14, and 28 days of curing were obtained with a Student’s t-test, which is a widely adopted statistical test that was developed by William Sealy Gosset in 1905. It is used to investigate significant differences between the means of two sets of data, with a significance level of 0.05 (α = 0.05). Several remarkable studies have used the t-test to assess statistically significant differences among experimental data [63,64,65]. Table 7 shows the variability of the CBR results and the effect of soaking on the CBR value after 7, 14, and 28 days of curing. It can be observed that the statistically significant differences between the soaked and unsoaked CBR values vanished as curing time increased. This may have been due to the hydration of SF causing the matrix of peat to be cemented after proper curing, thus reducing the strength-degrading effect of soaking.

**Table 7:** Statistical analysis results for the verification of the variable similarity of CBR values (soaked and unsoaked) of 7, 14, and 28 days of curing.

	7 Days	14 Days	28 Days
Mean	6.27	9.17	10.07
Variance	1.21	6.10	8.00
P (T ≤ t) one-tail	0.02	0.05	0.04
t Critical one-tail	2.13	2.13	2.13
P (T ≤ t) two-tail	0.04	0.10	0.09
t Critical two-tail	2.78	2.78	2.78
Statistically significant difference?	Yes (*p* < 0.05)	No (*p* > 0.05)	No (*p* > 0.05)

## 4. Conclusions

This study was aimed to quantify and assess the mechanical property variations of peat resulting from the incorporation of hydraulic binders (SF and OPC) through a systematic experimental procedure. In addition to the index and compaction characteristics, the experimental program included UCS and CBR tests for mechanical property and binder effectiveness assessments. The microstructural details were obtained with SEM. The derived results were analyzed, and the following conclusions were drawn.

1. Teluk Intan peat contains high water, organic, and fiber contents; is classified as an H_3_ level of humification that is acidic in nature; and possesses a low UCS value of 42.94 kPa.
2. Overall, the MDD values increased and the OMC reduces with increasing amounts of SF and OPC due to the involvement of hydraulic reactions.
3. Similar to MDD, the mechanical properties (UCS and CBR) of the stabilized peat were enhanced with increasing amounts of binders and longer curing periods. The effectiveness of these binders was acceptable for highway subgrade improvement after 28 days of proper curing.
4. Considering the targeted SDI of 7.03, the strength development by SF more rapid than that of OPC.
5. Both the hydraulic binders (SF and OPC) and their mixes enhanced strength but induced brittle failure after increasing the binder dosage.
6. The morphological studies revealed the hollow cavities/pores, spongy organic matter, flaky and loosely packed internal structure of the parent peat, which evolved into a compact matrix with strong interparticle bonds when using SF and OPC.
7. Effective statistical models were generated for the assessment of UCS and CBR (soaked and unsoaked) with increasing amounts of SF ranging from 10 to 20% and curing periods of 7, 14, and 28 days. Moreover, a strong correlation (R^2^ > 0.9) was observed between the UCS and CBR (soaked and unsoaked) of SF-stabilized peat.

## Figures and Tables

**Table 1:** Chemical compositions (weight percentages) of peat, SF, and OPC revealed by XRF analysis.

Oxides (% Weight)	Peat	Silica Fume	OPC
CO_2_	90.12	-	-
Al_2_O_3_	0.746	0.876	2.68
SiO_2_	6.64	91.0	12.3
CaO	0.355	1.20	75.5
Fe_2_O_3_	0.673	3.39	2.86
K_2_O	0.045	1.42	0.479
TiO_2_	0.020	0.013	0.231
SO_3_	0.942	0.352	1.32
MgO	0.142	0.409	1.80
P_2_O_5_	0.030	-	-
ZrO_2_	-	0.075	0.217
MoO_3_	0.24	0.052	1.29
MnO	-	0.205	0.411
ZnO	0.032	0.0292	0.0275
Total weight (%)	99.98	99.02	99.10

**Table 2:** Physical and chemical properties of silica fume.

Properties	Value/Description
Appearance	Ultrafine amorphous powder
Color	Gray, off-white
Odor	Odorless
pH @ 20 °C	6.0–9.0
Solubility (water)	Insoluble/slightly soluble
Solubility (organic solvents)	Insoluble/slightly soluble
Boiling point	No information
Melting point	1550–1700 °C
Bulk density	150–700 kg/m^3^
Specific gravity	2100–2300 kg/m^3^
Particle Size	0.4–0.5 μm

**Table 3:** Summary of the mix design.

Design Mixes	Peat	SF	OPC
Peat	100%	-	-
Peat and 10% SF	90%	10%	-
Peat and 15% SF	85%	15%	-
Peat and 20% SF	80%	20%	-
Peat and 15% OPC	85%	-	15%
Peat, 15% SF, and 15% OPC	60%	15%	15%
Peat, 5% SF, and 15% OPC	80%	5%	15%
Peat, 10% SF, and 10% OPC	80%	10%	10%
Peat, 15% SF, and 5% OPC	80%	15%	5%

**Table 5:** Essential indexed and compaction properties of Teluk Intan peat.

Characteristics	Values	Guidelines
Color	Blackish brown	-
Moisture content, %	224.18	ASTM D 2974-00 [20]
von Post humification	H_3_	Landva and Pheeney (1980) [23]
Specific gravity, *G_s_*	1.88	ASTM D 854–14 [24]
Liquid limit, LL (%)	64.4	ASTM D4318–10 [25]
Permeability, *k_v_* (m/sec)	5.86 × 10^−5^	ASTM D2434–68 [26]
pH (peat leachate) @ 20 °C	4.0	ASTM D4972–19
Ash content, AC (%)	19.13	ASTM D2974–00 [20]
Organic content, OC (%)	80.86	ASTM D2974–00 [20]
Fiber content, FC (%)	80.4	ASTM D 1997–91 [27]
**Compaction Properties**		
Optimum moisture content, OMC (%)	31.1	ASTM D698–12 [16]
Maximum dry density, MDD (kg/m^3^)	1130	ASTM D698–12 [16]
Unconfined compressive strength, UCS (kPa)	42.94	ASTM D2166/D2166M-16 [13]

**Table 6:** Relative ratings of CBR values for subgrade.

Material	CBR Value (%)	Rating/Remarks
Subgrade	<5	Very poor
Subgrade	5–10	Poor–fair
Subgrade	10–20	Fair–good
Subgrade	20–30	Very good

## Data Availability

Not applicable.
